# Constipation and Corn

**Published:** 1862-05

**Authors:** 


**CONSTIPATION AND CORN.**—A table-spoonful of coarse corn-meal (Indian) stirred in a glass of cold water, and drank quickly, on rising in the morning, has frequently had an excellent effect in keeping the system healthful and free. Living for a week wholly on sweet, fresh, pure milk, with corn-meal mush, has a most wholesome effect where there is headache, dullness, cold feet, and an indifferent appetite. It would be well for dyspeptic persons to use corn-meal more freely in their diet, preparing it in a great variety of ways, so that it may not pall on the taste. A delightful corn griddle-cake is thus made: Scald at night half the quantity of meal you are going to use, mix the other with cold water, having it the consistency of thick batter; add a little salt, and set it to rise; it will need no yeast. In the morning the cakes will be light and crisp. Skimmings, where meat has been boiled, is best for frying them with. Fry slowly.

The *American Agriculturist* says, that of a multitude of competitors, Mrs. James O'Brien, of Carrick, Pennsylvania, makes the best corn-bread thus: To two quarts of meal add one pint of bread-sponge; water sufficient to wet the whole; add half a pint of flour and a table-spoonful of salt; let it rise; then knead well for the second time, and place the dough in the oven, and allow it to bake an hour and a half.

